# Advances in Portable Biosensor-Based Test Kits for Pesticide Residue Screening in Agricultural Products: A Systematic Review

**DOI:** 10.3390/foods15081412

**Published:** 2026-04-17

**Authors:** Udomsap Jaitham, Wenting Li, Sumed Yadoung, Peerapong Jeeno, Xianfeng Cao, Ching Sian Zam, Surat Hongsibsong

**Affiliations:** 1 School of Health Sciences Research, Research Institute for Health Sciences, Chiang Mai University, Chiang Mai 50200, Thailand; udomsap_j@cmu.ac.th (U.J.); wenting_l@cmu.ac.th (W.L.); sumed.yadoung@cmu.ac.th (S.Y.); peerapong_jeen@cmu.ac.th (P.J.); xianfeng_cao@cmu.ac.th (X.C.); chingsianzam_1@cmu.ac.th (C.S.Z.); 2 Environmental, Occupational Health Sciences and Non-Communicable Diseases Center of Excellence, Research Institute for Health Sciences, Chiang Mai University, Chiang Mai 50200, Thailand

**Keywords:** pesticide residues, biosensors, rapid screening, nanomaterials, food safety, regulatory screening

## Abstract

Pesticide residues in food and agricultural products continue to constitute a significant concern for food safety, particularly when rapid decision-making is required across production and supply chains. Although chromatographic methods such as GC-MS and LC-MS/MS remain essential for confirmatory analysis, their dependence on central laboratories limits their applicability for field screening. Consequently, portable biosensor-based detection platforms have attracted increasing attention as rapid screening tools. This review synthesizes 26 peer-reviewed studies published between 2010 and 2025 on portable biosensor-based screening tools for pesticide detection in food and agricultural matrices, including electrochemical sensors, immunoassays, aptamer-based systems, paper-based lateral flow devices, and smartphone-assisted platforms. Given the heterogeneity of analytes, sensing mechanisms, and study designs, a narrative synthesis approach was applied. Overall, the evidence suggests a shift from laboratory-centered detection toward field-deployable technologies that may support preliminary screening within food safety monitoring frameworks. Paper-based lateral flow assays are widely reported as deployable formats, while electrochemical and affinity-based platforms are often positioned as intermediate solutions for mobile or semi-controlled testing environments. However, most platforms remain at the proof-of-concept or early validation stage, and challenges related to matrix interference, long-term stability, reproducibility, standardization, and large-scale implementation persist. This review highlights the potential role of portable biosensor technologies as complementary tools within tiered food safety monitoring systems and outlines key priorities for further development before wider regulatory integration can be considered.

## 1. Introduction

In modern agricultural production, pesticides are widely used to control pests and diseases and ensure crop yields. However, improper use or excessive application of pesticides can lead to their residues in food and environmental media, posing potential risks to human health and ecosystem security [1,2]. Numerous epidemiological and toxicological studies have shown that long-term or low-dose exposure to various commonly used pesticides, especially organophosphates, neonicotinoids, and pyrethroids, is closely associated with neurotoxic effects, endocrine disruption, and an increased risk of chronic diseases [3]. Given that pesticides can continuously enter the human body through the food chain and environmental media, systematic and effective monitoring of pesticide residues in food and agricultural products is considered a key measure to ensure food safety and public health [2].

Currently, gas chromatography-mass spectrometry (GC-MS) and liquid chromatography-tandem mass spectrometry (LC-MS/MS) are still widely used and regarded as reference methods for pesticide residue analysis in food due to their high sensitivity and reliable quantitative performance [4,5]. However, these methods typically rely on complex sample pretreatment procedures to effectively extract target pesticides and remove matrix interference [6], and the expensive equipment, high maintenance costs, and requirement for skilled technicians result in long detection cycles [7]. Therefore, these traditional laboratory methods struggle to meet the demands for rapid decision-making in market regulation, field monitoring, or supply chain stages, and their accessibility and efficiency are significantly limited in resource-constrained areas or scenarios requiring frequent inspections [8].

Against this backdrop, portable pesticide detection technologies for field applications have gradually gained attention. To ensure conceptual clarity across diverse studies, key terms used in this review are defined as follows. “Portable” and “field-deployable” refer to detection systems that can be operated outside centralized laboratory settings with minimal infrastructure and technical requirements. “Rapid screening” refers to analytical approaches designed for timely, preliminary detection rather than confirmatory quantification. “Biosensor-based test kits are devices that incorporate biological recognition elements, such as enzymes, antibodies, or aptamers, to generate measurable signals for on-site or near-site pesticide detection. Over the past decade, various rapid detection platforms based on biosensor principles have emerged, including electrochemical sensors, immunoassays, aptamer sensors, paper-based lateral chromatography test strips, and smartphone-assisted detection systems [9,10,11]. These platforms typically use enzymes, antibodies, or aptamers as recognition elements, converting chemical/biological interactions such as target binding into measurable signals, thereby enabling rapid response and on-site screening of pesticide residues [9,12].

At the same time, the introduction of nanomaterials provides a key technological foundation for improving the detection performance of portable biosensors. Metal nanoparticles, carbon-based materials, semiconductor nanostructures, and nanozyme systems have been widely applied to signal amplification, radiometric readouts, and multimodal detection designs, thereby significantly reducing the detection limits while maintaining ease of operation [13,14]. For example, sensing platforms based on nanozymes or single-atom catalytic systems have achieved detection sensitivities close to or below the relevant regulatory limits in several studies, demonstrating their significant potential for rapid screening and field applications [15,16].

Previous review articles on pesticide detection technologies have primarily focused on sensor chemistry, nanomaterial development, analytical performance, and general detection strategies. However, these reviews have less frequently addressed the practical role of portable biosensor-based test kits in food and agricultural matrices, particularly in terms of field-readiness, usability across different platform types, and their integration into tiered regulatory screening workflows.

In addition, differences in detection limits, sample matrices, and validation strategies across studies make cross-platform comparisons challenging [13]. While some detection systems demonstrate excellent analytical performance under laboratory conditions, their applicability, operational complexity, and long-term stability in real-world environments remain insufficiently evaluated [17]. Furthermore, there is currently no clear consensus on the functional positioning of these portable detection platforms within food safety regulatory systems, particularly regarding their roles as primary screening tools versus complementary or confirmatory methods [18].

This review aims to address existing gaps by comparatively synthesizing portable biosensor-based screening tools according to device format, biorecognition strategy, analytical performance, and operational suitability for decentralized pesticide residue detection. Instead of treating all biosensor studies as analytically equivalent, this review emphasizes how factors such as portability, validation practices, and workflow complexity influence their realistic applicability in food safety monitoring.

Based on this, the present systematic review aims to comprehensively review and compare the technological advancements and application performance of portable biosensors in pesticide residue detection in food and agricultural products in recent years. By integrating 26 peer-reviewed studies published between 2010 and 2025, the review systematically analyzes the analytical performance, recognition strategies, and nanomaterial-based signal amplification mechanisms of different sensing platforms, and evaluates their technological strengths and limitations in terms of sensitivity, operational complexity, and field deploy ability.

## 2. Materials and Methods

### 2.1. Literature Search

This review followed a structured literature search strategy based on the PRISMA 2020 principles to identify peer-reviewed studies related to biosensor-based and nanomaterial-assisted rapid test kits for pesticide detection in food and agricultural products.

The literature search was conducted across three major scientific databases: PubMed, Scopus, and ScienceDirect. Searches were performed independently in each database, and retrieved articles were combined. The search covered publications from January 2010 to December 2025. Keyword combinations related to pesticides and agrochemicals, biosensor-based detection technologies, nanomaterial-assisted sensing strategies, and rapid or portable testing formats for food and agricultural products were used.

**Figure 1:**
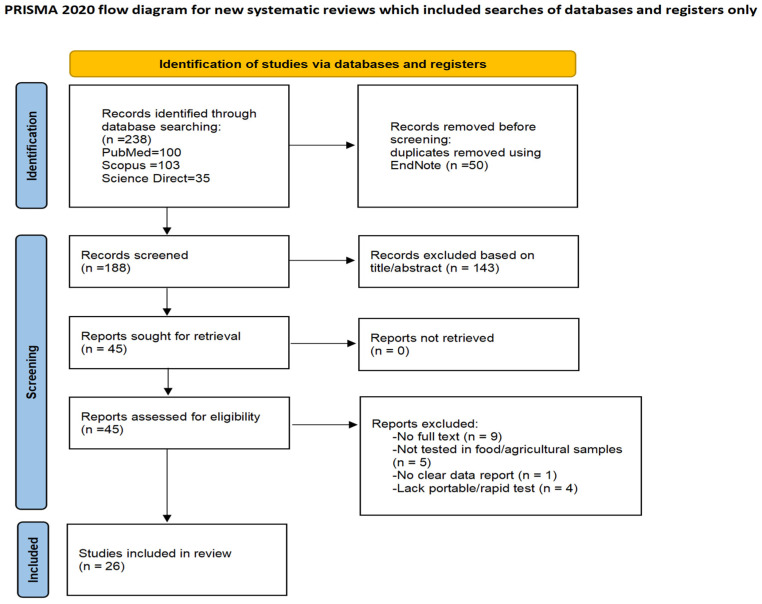
PRISMA 2020 flow diagram of the study selection and inclusion process.

A total of 238 records were retrieved from three databases: 100 from PubMed, 35 from ScienceDirect, and 103 from Scopus. After removing 50 duplicate records using EndNote 20 (Clarivate, London, UK), 188 studies remained for title and abstract screening. Following the exclusion of evidently unrelated articles through automated relevance filtering and manual review, 45 studies were assessed in full text. After applying the predefined inclusion criteria, it ultimately included 26 studies. The study identification and selection process is summarized in the PRISMA 2020 flow diagram (Figure 1). The completed PRISMA 2020 checklist is provided as Supplementary Material S1 [19].

### 2.2. Inclusion and Exclusion Criteria

The eligibility of studies was determined to ensure that all included research aligned with the scope of the biosensor-based and nanomaterial-assisted rapid detection of pesticide residues in agricultural products. Studies were included if they met the following criteria: (i) original peer-reviewed research articles published in English between January 2010 and December 2025; (ii) studies involving the development or application of biosensors, immunosensors, aptasensors, enzymatic systems, nanozyme-based platforms, or other nanotechnology-assisted rapid test kits; (iii) research focused on the detection of pesticides or agrochemicals in agricultural products, including food commodities, crops, fruits, vegetables, or related matrices; (iv) use of biological recognition or nanomaterial-based components (e.g., nanoparticles, nanozymes, molecularly imprinted polymers, aptamers, antibodies, acetylcholinesterase) as part of the sensing or signal amplification mechanism; (v) incorporation of rapid, on-site, portable, point-of-care, or field-deployable detection approaches (e.g., lateral flow assays, paper-based devices, strip tests, electrochemical portable sensors); (vi) reporting of analytical performance parameters, such as limit of detection (LOD), linear range, sensitivity, recovery rates in spiked or real samples, and/or validation against reference laboratory methods (HPLC, GC, LC-MS/MS); (vii) relevance to pesticide classes of regulatory and food-safety concern, including but not limited to organophosphates, carbamates, neonicotinoids, herbicides, and fungicides.

Studies were excluded if they were (i) review papers, conference abstracts, theses, commentaries, book chapters, or non-original research formats; (ii) studies lacking biosensor or nanotechnology-based elements (e.g., exclusively conventional chromatographic or spectroscopic analysis without a rapid/portable component); (iii) research focused solely on environmental matrices (soil, water, or air) without relevance to agricultural or food products; (iv) insufficient methodological or analytical reporting, including studies with no LOD, no validation data, or unclear detection outcomes; (v) non-English publications or studies outside the defined timeframe; (vi) articles for which the full text was not accessible.

### 2.3. Data Extraction

To ensure consistency in data extraction and to minimize extraction bias, data extraction was performed independently by two reviewers. A structured Microsoft Excel database (Microsoft 365, Microsoft Corporation, Redmond, WA, USA) database was developed specifically for this review, and all relevant information from the included studies was recorded using predefined data fields. For each eligible article, bibliographic details (author, year of publication), target pesticide, and sample matrix (e.g., fruit, vegetable, tea, grain, or processed agricultural product) were recorded. Sensor-specific characteristics were also extracted, including the recognition element used (acetylcholinesterase, aptamer, antibody, nanozyme, or molecularly imprinted polymer), the type of sensing platform (immunosensor, aptasensor, electrochemical device, colorimetric strip, or lateral flow assay), and the nanomaterial composition involved in signal transduction or amplification (e.g., metal nanoparticles, metal–organic frameworks, carbon-based nanomaterials, or nanocomposite hybrids). In addition, studies were grouped into platform categories based on device format and field deployment characteristics (e.g., electrochemical sensors, paper-based lateral flow assays, smartphone-assisted systems), rather than solely on sensing mechanisms, to better reflect their practical application in field-based pesticide screening.

Analytical performance parameters were systematically recorded, with particular focus on the reported limit of detection (LOD), linear detection range, sensitivity values, validation techniques, and recovery rates in real or spiked agricultural samples. When available, cross-validation using reference laboratory methods such as HPLC, GC, LC-MS/MS, GC-MS, and ELISA was documented to assess the reliability of each platform relative to the reference methods under applied conditions. To facilitate meaningful comparisons between different studies, all reported LOD values were converted to a uniform unit (µg/kg) based on the information provided. Studies that reported values in unconventional units were re-evaluated by both reviewers, and conversions were only performed when original calibration or unit references were explicitly stated. To ensure comparability across studies, data harmonization focused on standardizing analytical performance metrics, particularly LOD values, and consistently documenting validation approaches and sample matrices, rather than assuming direct equivalence between different sensing systems.

Any disagreements during data extraction were resolved through re-examination of the original articles and their Supplementary Materials. If essential analytical information such as LOD, linear range, or validation reference data was unclear, the study was re-evaluated to confirm inclusion eligibility based on methodological completeness. In cases where pesticide groups were screened but not quantified, the study was recorded qualitatively but flagged as having limited numerical comparability.

Due to substantial heterogeneity among the included studies, no formal meta-analysis was conducted. Specifically, the studies differed in target analytes (including the number and classes of pesticides assessed), sample matrices (e.g., fruits, vegetables, grains, beverages, and water-associated matrices), sensing mechanisms and platform formats (e.g., electrochemical, optical, enzymatic, paper-based, and smartphone-assisted systems), and validation approaches. In addition, the reported analytical performance metrics were not fully comparable across studies because the detection limits were originally expressed in different units, and some studies reported qualitative or semi-quantitative screening outcomes rather than directly comparable quantitative endpoints. These differences limited the feasibility of combining results through formal quantitative synthesis. Instead, a narrative synthesis approach was adopted to summarize platform characteristics, performance trends, and the applicability of different biosensor configurations for on-site pesticide detection in agricultural products. This approach also allowed for a clearer differentiation between proof-of-concept laboratory devices and field-ready platforms with reported use in real sample conditions.

### 2.4. Evaluation of Methodological Quality and Risk of Bias

Because the included studies primarily focused on the development and analytical evaluation of biosensor-based detection platforms, conventional risk-of-bias assessment frameworks used in clinical or epidemiological reviews were not directly applicable. Instead, the methodological quality and potential sources of bias in each study were evaluated using a structured qualitative assessment framework tailored to analytical performance and validation practices.

Particular attention was given to reported limits of detection, linear ranges, recovery in real or spiked food samples, reproducibility indicators, and whether the results were cross-validated against established chromatographic reference methods. These parameters were used to assess the analytical reliability and field applicability of the reported rapid pesticide detection systems.

To standardize this evaluation, studies were qualitatively stratified based on three critical domains of analytical rigor:

(i) Validation strategy: ranging from chromatographic confirmation (e.g., LC-MS/MS, GC-MS, or HPLC comparison), recovery-based evaluation in real matrices, to studies lacking external validation;
(ii) Matrix complexity: categorized according to the testing environment (e.g., buffer systems, spiked samples, or complex real agricultural matrices), recognizing that matrix effects significantly influence biosensor performance;
(iii) Reproducibility and precision: assessed based on the reporting of replicate measurements, relative standard deviation (RSD), or inter-assay consistency.

Each domain was qualitatively categorized (e.g., high, moderate, or low) based on the level of validation and reporting completeness, allowing for consistent comparison across studies with heterogeneous methodologies. This categorization was applied uniformly across all included studies to support transparent evaluation of methodological robustness.

Rather than generating a numerical risk-of-bias score, this matrix- and validation-focused framework served as a pragmatic quality assessment approach, enabling differentiation between proof-of-concept studies and more application-oriented platforms that reported validation under realistic conditions. Studies lacking sufficient methodological detail across these domains were considered to have a higher risk of analytical bias and were interpreted with caution.

It should be noted that due to the heterogeneity in sensing mechanisms, target analytes, and experimental conditions, this qualitative framework is intended to support transparent interpretation rather than direct quantitative comparison of study quality.

## 3. Results

This review included 26 studies, covering research published in multiple countries between 2010 and 2025. Collectively, these studies illustrate a clear methodological shift in pesticide residue analysis from conventional, laboratory-bound chromatographic instrumentation toward portable, field-deployable biosensor platforms intended for rapid screening. Most studies focused on agricultural substrates, including fruits, vegetables, grains, tea, and processed plant materials, reflecting the actual needs of food inspection and supply chain monitoring. Across studies, rapid biosensing technologies, particularly those incorporating nanomaterial-assisted signal amplification, have been reported to achieve lower detection limits, in some cases approaching those of chromatographic methods.

For clarity of presentation, the results are organized according to two complementary analytical dimensions: (i) the primary sensing platforms employed for pesticide detection (Section 3.2), and (ii) the nanomaterial-based enhancement strategies used to improve analytical performance and on-site applicability (Section 3.4). This structure distinguishes between platform design and signal enhancement strategies. While several platforms have been applied in rapid screening contexts, most are used alongside confirmatory laboratory methods rather than as standalone analytical tools.

### 3.1. Overview of Included Studies

A total of 26 studies met the inclusion criteria and were included in the final analysis. These studies, published between 2010 and 2025, originated from multiple countries (Table 1). The majority of studies were conducted in China (*n* = 19), while the remaining studies were from Iran, India, Indonesia, Italy, South Korea, and the United States. These studies were conducted across multiple regions, indicating that research on portable biosensing platforms has been reported in diverse geographical settings.

The studies deleted included a wide range of pesticide classes, with a primary focus deleted on organophosphorus pesticides. Among the most commonly studied analytes include chlorpyrifos, malathion, diazinon, triazophos, and parathion. In contrast, neonicotinoid pesticides (e.g., acetamiprid and thiamethoxam) were less frequently examined, and comparatively fewer studies addressed fungicides and herbicides. This imbalance may reflect a research focus on pesticide classes associated with acetylcholinesterase inhibition mechanisms, which are more commonly targeted in biosensor development.

Regarding sample matrices, the majority of studies evaluated detection in agricultural produce, including fruits, vegetables, grains, and tea, often with additional validation in water or processed food samples. While many studies employed real agricultural matrices, the complexity of experimental conditions varied significantly, ranging from simple spiked samples to heterogeneous real food systems. This variability may influence the comparability of reported performance and limit the generalizability of findings across different food matrices.

In terms of sensing platforms, lateral-flow immunoassays (LFAs) and smartphone-assisted paper-based systems were the most commonly reported formats, likely due to their suitability for rapid and decentralized screening. The second most frequently reported category comprised electrochemical platforms based on screen-printed electrodes (SPE/SPCE) and portable potentiostats. Additional methods included reader-assisted strip systems and optical or colorimetric assays, some of which incorporated advanced signal amplification techniques, such as SERS or nanozyme catalysis.

Across various platform types, enzymatic inhibition, particularly acetylcholinesterase-based detection, was frequently used as a recognition strategy, especially for organophosphorus and carbamate pesticides. Immunosensor and aptasensor designs were also commonly reported, alongside affinity-based recognition approaches (such as antibody- and aptamer-based systems).

Detection limits among studies were widely different because of the variation of sensing mechanisms, target analytes, and experiment conditions. SERS-, plasmonic enhancement-, or nanozyme catalyst-based systems have been reported to achieve low detection limits, with a few studies reporting sub-ng/mL performance and detection limits below 0.01 µg/kg [21,31,39], Conversely, earlier-generation colorimetric inhibition assays generally showed higher detection limits in the range of µg/kg to mg/kg [27,38]. Electrochemical and affinity-based systems typically exhibited intermediate sensitivity, often relying on electrode modification and signal amplification strategies.

Despite this variability, many platforms reported detection performance within ranges relevant to regulatory maximum residue limits (MRLs) when tested in agricultural matrices. More than half of the included studies reported chromatographic validation (e.g., LC–MS/MS, GC, or HPLC comparison), which may support the analytical reliability of these systems under the reported conditions [20,32,43].

The methodological strengths of the studies are included in Table 2, as well as the validation strategies, complexity of the matrices, and reproducibility. Studies that employed chromatographic validation and tested actual agricultural matrices were more frequently classified as of greater methodological strength. Conversely, those studies that depended on recovery-based evaluation or those with limited amounts of validation were associated with less robustness, especially those where the measurements of reproducibility were inadequately reported.

Altogether, the findings indicate the increased attention to portable, rapid, and primarily semi-quantitative methods for pesticide detection. These systems have mostly been developed to be used in first line screening applications in the food supply chain with different levels of analytical performance and methodological strength of the different platform types.

### 3.2. Platform-Based Classification of Biosensor Systems

To provide a systematic overview of portable pesticide biosensing technology, the included studies were categorized according to five main device platforms, as summarized in Table 3. This platform-based classification reflects the physical implementation and practical usage of sensing systems. Within each category, key sensing mechanisms, recognition strategies, and operational characteristics are comparatively discussed to highlight common patterns and differences across platforms and to better understand how these factors may influence overall system performance.

#### 3.2.1. Electrochemical Platforms

One of the most commonly reported platform types among the included studies was electrochemical biosensors. Most systems utilized disposable screen-printed electrodes (SPEs or SPCEs) and generated analytical signals through changes in current or potential resulting from enzymatic inhibition or affinity-based binding events. These platforms are typically integrated with portable potentiostats, enabling rapid signal transduction without the need for conventional bench-top instrumentation and facilitating in situ screening applications.

Across the various agricultural matrices, including cabbage, leek, tomato, and mixed fruit samples, electrochemical biosensors are generally applied using relatively simple sample preparation approaches, such as extraction or dilution-based protocols [35,36,45]. A common pattern observed across studies is the reliance on enzyme inhibition, particularly acetylcholinesterase-based detection, as the primary recognition mechanism. However, in samples with high pigment or fiber content, these systems may be susceptible to matrix effects that interfere with signal generation and reduce analytical reliability. Alternatively, antibody- and aptamer-based electrochemical configurations represent a second major design category, offering improved selectivity through specific molecular recognition. From an application perspective, electrochemical biosensors are primarily used for rapid screening rather than serving as standalone confirmatory analytical tools.

To achieve rapid and sensitive signal transduction, the reviewed electrochemical platforms employ a range of interrogation techniques, primarily differential pulse voltammetry (DPV), square wave voltammetry (SWV), and electrochemical impedance spectroscopy (EIS). Voltametric methods, such as DPV and SWV, are widely used due to their ability to minimize background charging currents, thereby enhancing sensitivity for detecting electroactive species generated during enzymatic reactions or direct pesticide oxidation. In contrast, EIS provides a label-free detection approach by monitoring changes in interfacial charge transfer resistance upon the binding of target analytes to immobilized recognition elements, making it particularly suitable for affinity-based sensing strategies.

A key advancement in these platforms is the development of surface modification strategies aimed at overcoming the intrinsic limitations of bare screen-printed electrodes, such as slow electron transfer kinetics and susceptibility to electrode fouling. The incorporation of nanostructured materials such as metal–organic frameworks (MOFs), carbon nanotubes (CNTs), and metallic nanoparticles significantly enhances electrochemical performance by increasing the effective surface area and facilitating electron transfer. Additionally, these materials also enable the high-density immobilization of biorecognition elements, thereby improving sensitivity and signal stability.

Moreover, nanostructured electrode interfaces have been shown to partially mitigate electrode fouling by providing structural and electrostatic barriers against matrix interferents, such as proteins, lipids, and plant-derived pigments. This effect can help maintain signal stability during the analysis of complex food samples. However, despite these advancements, surface fouling and matrix-induced variability remain significant challenges, particularly under real-world testing conditions where sample composition is highly heterogeneous.

#### 3.2.2. Paper-Based Platforms (LFA)

Paper-based platforms, especially lateral flow assays (LFA), are widely employed in the included studies due to their ease of use, low material cost, and suitability for deployment in decentralized field settings. In practice, these platforms generally require minimal sample preparation and offer short assay times, typically within minutes, which facilitates rapid decision-making in field conditions [28,41]. Recent developments have incorporated nanomaterial-based signal enhancement strategies to improve robustness for initial screening in complex food matrices. Across studies, a consistent pattern is that these platforms are primarily applied for rapid, preliminary detection with minimal reliance on instrumentation, although the level of simplification may vary depending on system design and target analytes.

#### 3.2.3. Reader-Assisted Strip Platforms

To overcome the limitations of naked-eye visual interpretation, several studies employed reader-assisted strip platforms. These systems utilize handheld or portable instruments to quantify the colorimetric or fluorescent signals generated on paper-based strips or LFAs. Signal enhancement strategies, including dual-mode readouts and machine learning-assisted interpretation, have been increasingly incorporated to improve analytical robustness and reduce user subjectivity during result interpretation [21,46]. This category maintains the operational convenience of strip-based tests while enabling semi-quantitative signal interpretation through reader-assisted measurement.

#### 3.2.4. Smartphone-Assisted Systems

Smartphone-assisted biosensing platforms have been increasingly reported as portable systems for semi-quantitative pesticide screening. These platforms integrate optical or electrochemical signal outputs with smartphone-based image acquisition, data processing, or algorithm-assisted quantification, enabling on-site analysis without dedicated laboratory instrumentation. Early smartphone-based systems primarily relied on RGB or grayscale image capture, whereas more recent implementations employed ratiometric fluorescence imaging, plasmonic signal modulation, or algorithm-assisted lateral flow readouts to enhance consistency and reduce operator-dependent variability [20,41]. In summary, smartphone-assisted systems primarily function as signal interpretation and quantification tools that support field-based application of portable biosensors.

#### 3.2.5. Optical and Colorimetric Platforms

Standalone optical and colorimetric platforms typically rely on solution-based enzymatic reactions, plasmonic effects, or portable optical instruments without the specific integration of paper strips or smartphones. Although early colorimetric systems were typically limited by high detection limits, optical sensing platforms developed in recent years have been increasingly applied in preliminary residue screening. For instance, platforms employing surface-enhanced Raman scattering (SERS) or portable fluorescence readouts have been reported to achieve low detection limits [28]. Overall, simple colorimetric assays in this category are mostly used for rapid presence/absence screening, whereas advanced optical platforms have been reported to achieve lower detection limits in portable formats.

### 3.3. Biorecognition Strategies in Portable Platforms

While the previous section categorizes sensors by their physical hardware, the analytical specificity of these portable platforms is driven by their biological recognition elements. Importantly, immunosensors and aptasensors in this context refer to affinity recognition strategies rather than standalone device formats, and are implemented across multiple platform architectures.

Immunosensor-based methods that use antibodies as screeners were widely reported to screen pesticide residues, especially neonicotinoid and organophosphate pesticides. They were often paired with electrochemical, fluorescence, or surface-enhanced Raman scattering (SERS) outputs and have been reported to enable selective detection across a range of agricultural matrices [32,45].

In the included studies, aptamer-based biosensors were frequently reported as alternative affinity recognition strategies. These platforms were implemented in lateral flow, electrochemical, and optical configurations and were commonly validated using chromatographic reference methods or recovery-based experiments [35,39]. Recent studies have increasingly incorporated multiplex detection designs, allowing for the simultaneous screening of multiple pesticide residues within a single assay [25,42]. Across studies, immunosensor- and aptasensor-based strategies were associated with selective target recognition and have been applied across diverse platform configurations. Table 3 summarizes the primary mechanisms, key characteristics, and field deployment considerations of enzymes, antibodies, and aptamers utilized in the reviewed platforms.

### 3.4. Analytical Performance Comparison Across Platforms

To enable a standardized comparison of analytical performance across portable pesticide detection platforms, all reported limits of detection (LODs) were converted to a common unit (µg/kg). This conversion is important for enabling comparison, as original studies report sensitivity in different metrics (e.g., molarity, ng/mL, ppb, or ppm). Standardizing to µg/kg helps reduce these discrepancies and facilitates alignment with regulatory maximum residue limits (MRLs) established for agricultural commodities. However, it is important to emphasize that LOD values reported across studies are influenced by multiple factors, including differences in target analytes, sample matrices, sensing mechanisms, and validation protocols. Therefore, the standardized LOD values presented in this analysis should be interpreted as descriptive indicators of platform performance rather than direct comparative benchmarks. In particular, cross-platform comparisons may not fully reflect real-world applicability unless they are considered within the context of matrix effects, validation rigor, and operational conditions.

Following unit standardization, the sensors were grouped by their primary sensing platform. Given the inherent differences in target analytes, recognition elements, and matrix complexities, the sensitivity data exhibited significant variance. Consequently, the median LOD was selected as the primary metric to characterize the typical performance of each platform category, rather than relying on mean averages or best-case (minimum) values. While best-case values highlight the theoretical ceiling of a specific technology, the median provides a more realistic, statistically robust benchmark that resists skewing from extreme outliers or highly optimized, proof-of-concept laboratory conditions.

To maintain the integrity of this comparative analysis, multi-modal systems (e.g., platforms employing simultaneous colorimetric and fluorescent readouts) and laboratory-assisted platforms (e.g., those requiring benchtop equipment for sample preparation or signal interpretation) were explicitly excluded from this specific median calculation. This exclusion helps prevent data double-counting and supports a more accurate reflection of the capabilities of field-deployable and portable systems.

**Figure 2:**
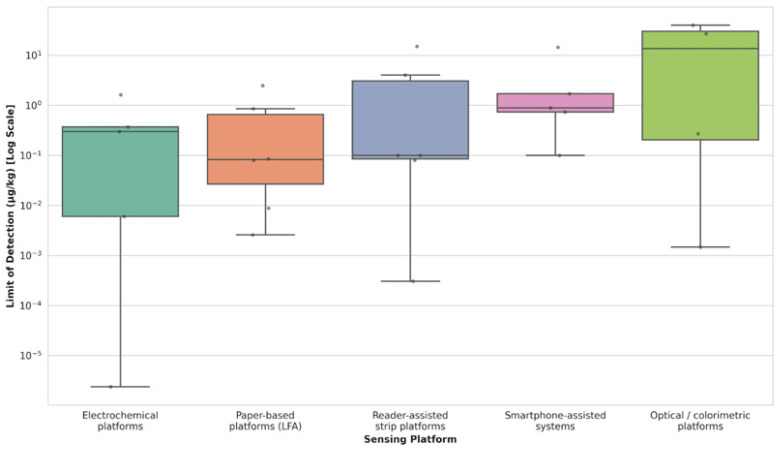
Distribution of limits of detection (LOD) across five portable pesticide sensing platforms. The dots represent individual data points (LOD values) extracted from the included studies for each platform.

These standardized metrics are presented in two complementary formats to provide a more comprehensive evaluation. Table 4 summarizes the qualitative operational characteristics, including typical validation approaches and relative field deployability. Figure 2 visualizes the statistical distribution of LOD values across platforms. Using a box-and-whisker plot, Figure 2 illustrates the median sensitivity, performance consistency (interquartile range), and variability within each platform category.

Electrochemical platforms (*n* = 5) exhibited the widest dynamic range of detection limits among all categories shown in Figure 2. This platform was associated with the lowest reported LOD in the reviewed literature (2.37 × 10^−6^ µg/kg) [39]. The broad interquartile range (IQR) indicates that analytical performance varies considerably depending on fabrication techniques, such as electrode surface modification and recognition element selection. This pattern suggests that electrochemical sensors can achieve low detection limits under optimized conditions; however, performance may vary substantially across studies due to differences in fabrication strategies and experimental conditions, which may limit direct comparability [29].

Paper-based lateral flow assays (LFA) (*n* = 6) exhibited a distinct performance profile, featuring the lowest median LOD (0.083 µg/kg) among fully field-deployable platforms and a relatively compact distribution. This narrower clustering of data points suggests relatively consistent performance across studies. For instance, a dual-mode LFA reported by [21] detected neonicotinoids at 0.12 µg/kg. Multiple studies reported detection limits within regulatory-relevant concentration ranges, suggesting that LFAs can achieve detection performance aligned with typical screening thresholds, although applicability may vary depending on target analytes and matrix conditions [44].

Reader-assisted strip platforms (*n* = 6) showed a performance distribution comparable to standard LFAs, with a median LOD of approximately 0.10 µg/kg. This category reflects a balance between analytical sensitivity and improved interpretation through simple instrumental readouts. The data spread ranged from low detection limits (3.0 × 10^−4^ µg/kg) reported by [31] to higher limits in earlier designs. This variability can be attributed to differences in reader technologies employed, where advanced fluorescence readers were associated with improved sensitivity compared to simple handheld colorimeters [47].

Smartphone-assisted detection systems (*n* = 5) exhibited a median LOD of approximately 0.89 µg/kg. In these platforms, optical or electrochemical sensing elements are coupled with smartphone-based image acquisition and algorithmic quantification, enabling semi-quantitative analysis without dedicated laboratory instrumentation. The distribution indicates that while these systems effectively bridge the gap between qualitative screening and instrumental analysis, their sensitivity is often limited by ambient lighting conditions and camera sensor quality. Representative studies have reported field-oriented applicability, such as the aptasensor developed by [42], which achieved an LOD of 0.73 µg/kg. Although these systems generally exhibit higher detection limits (i.e., lower sensitivity) than some electrochemical platforms, their performance may be interpreted in the context of operational simplicity and field applicability [41].

Finally, optical and colorimetric platforms (*n* = 4) exhibited the highest median LOD (1.0 µg/kg) and significant variability, with detection limits extending up to 40 µg/kg, as seen in the work of [38]. Colorimetric methods typically report higher detection limits because they prioritize ease of operation, low equipment dependence, and rapid visual interpretation over the pursuit of absolute analytical sensitivity. The elevated position of the box plot suggests limitations associated with reliance on visual readouts, indicating that these platforms are generally more suitable for rapid qualitative screening applications, particularly where ease of use and minimal equipment are prioritized over ultra-trace sensitivity [27].

**Figure 3:**
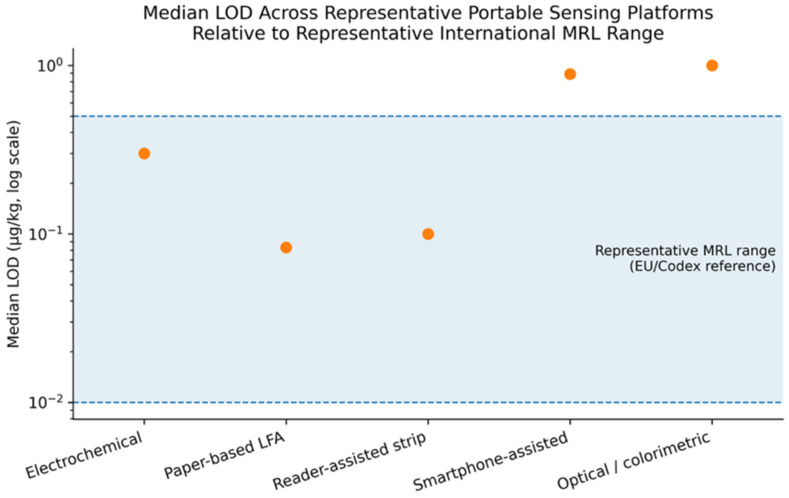
Median limits of detection (LOD) across representative portable sensing platforms relative to a representative international maximum residue limit (MRL) range derived from EU and Codex reference frameworks. LOD values are shown on a logarithmic scale. The shaded band indicates a representative regulatory range used for contextual interpretation rather than compound-specific comparison. The dots represent the calculated median LOD value for each respective sensing platform.

To provide a contextual interpretation of analytical sensitivity, Figure 3 presents the relationship between representative limits of detection (LOD) across different sensing platforms and typical international maximum residue limit (MRL) ranges.

As shown in Figure 3, the reported LOD values span several orders of magnitude across different platform categories. Paper-based lateral flow assays (LFAs) and reader-assisted strip platforms generally exhibit detection limits within or near the representative MRL range (approximately 0.01–0.5 mg/kg), indicating their potential suitability for preliminary screening applications. Electrochemical platforms also demonstrate LOD values within this range, although variability is observed across studies.

In contrast, smartphone-assisted and colorimetric platforms tend to exhibit higher LOD values, with some exceeding the upper bound of the representative MRL range. This suggests that under certain conditions, their sensitivity may be insufficient for detecting residues at or below the regulatory thresholds.

Overall, this comparison highlights that analytical sensitivity varies considerably among sensing platforms and should be interpreted in relation to regulatory thresholds rather than as an absolute measure of performance. Importantly, the MRL values presented here serve as representative reference ranges derived from international regulatory frameworks and are not intended as compound-specific thresholds.

### 3.5. Nanomaterial-Based Strategies for MRL-Relevant Signal Amplification

In recent years, nanomaterials have been widely used as signal amplification elements in portable pesticide detection platforms and are associated with improved detection performance. Rather than focusing solely on material novelty, many studies emphasize the functional role of these materials in achieving detection limits relevant to the regulatory maximum residue limits (MRLs) while maintaining operational simplicity suitable for on-site screening. A comprehensive summary of these nanomaterial-based signal amplification strategies, including their primary functional contributions and field deployment suitability, is presented in Table 5.

#### 3.5.1. Metal-Based Nanomaterials

Metal and metal-based nanomaterials represented one of the most widely applied amplification elements across the reviewed platforms. Noble metal nanoparticles, especially gold nanoparticles (AuNPs), are widely used in paper-based, colorimetric, electrochemical, and smartphone-assisted detection systems due to their combination of plasmonic properties, catalytic activity, and electrical conductivity.

In lateral flow and paper-based formats, AuNPs have been used to provide visually distinguishable or reader-assisted signal enhancement through localized surface plasmon resonance (LSPR), with reported detection limits reaching sub-µg/kg levels for neonicotinoid and fungicide residues in complex food matrices [21,44]. Smartphone-assisted LSPR systems further supported semi-quantitative interpretation without dedicated optical instruments, maintaining detection performance within regulatory-relevant ranges [41].

Metal nanostructures were also applied in fluorescence and surface-enhanced Raman scattering (SERS) platforms, where electromagnetic enhancement has been reported to support lower detection limits for multiple pesticide residues [26,32]. However, the reliance on auxiliary optical components somewhat diminishes the applicability of such systems in fully decentralized screening scenarios.

Overall, across studies, metal-based nanomaterials were primarily used as signal amplification elements that contributed to improved sensitivity in field-deployable systems, helping to reduce the gap between simple screening assays and laboratory-based analytical methods.

#### 3.5.2. Carbon-Based and Semiconductor Nanomaterials

Carbon-based and semiconductor nanomaterials were frequently reported across studies in optical and radiometric sensing platforms, particularly in smartphone-assisted and paper-based detection systems. Carbon dots and semiconductor nanoparticles have been used to provide photostable and tunable fluorescence signals, supporting portable and image-based quantification.

For example, carbon dot-based fluorescent sensors enabled smartphone-integrated detection of pyrethroid residues in tea and fruit matrices, achieving detection limits at the µg/kg level with chromatographic validation [20]. Semiconductor nanomaterials, especially upconversion nanoparticles (UCNPs), were widely used in ratiometric fluorescence platforms, improving signal robustness under ambient light conditions and enabling detection limits between 0.08 and 1.71 µg/kg for chlorpyrifos and neonicotinoids [22,37].

Although some hybrid semiconductor systems have been reported to achieve very low detection limits, across studies, these systems were primarily associated with improvements in signal stability and interpretability, rather than consistently achieving ultra-trace sensitivity.

#### 3.5.3. Nanozyme and Hybrid Nanocomposites

The use of nanozymes in amplification strategies was used more in the application of enzyme inhibition and affinity-based assays, especially to enhance sensitivity and stability of operations. Single-atom and bimetallic nanozymes that had peroxidase-like activity were utilized either as replacements to natural enzymes or catalytic signal amplifiers in lateral flow and immunoassay systems.

Representative studies have reported that nanozyme-assisted platforms can achieve detection limits below or close to relevant MRLs for organophosphate pesticides while maintaining compatibility with portable workflows [24,43]. Hybrid nanocomposite systems combining plasmonic, fluorescent, or catalytic components further enabled multi-modal or algorithm-assisted readout, enhancing robustness in complex food matrices [23,32].

Although many hybrid systems demonstrate strong analytical performance, their more complex workflows may limit their overall portability. Therefore, their application in food safety monitoring may be more appropriate for targeted screening or secondary testing applications, rather than routine large-scale field deployment.

Overall, across the reviewed studies, nanozyme and hybrid nanocomposite systems were primarily used as signal amplification strategies that contributed to achieving MRL-relevant sensitivity and improved signal robustness, although their performance and applicability may vary depending on system complexity and operational requirements.

### 3.6. On-Site and Field Applicability

Across the included studies, differences were observed in the field applicability of biosensor-based pesticide detection systems. Paper-based lateral flow assays (LFAs), reader-assisted strip platforms, and smartphone-assisted systems were most frequently applied in on-site and field-based settings. These platforms are associated with minimal sample preparation, short analysis times, and limited auxiliary equipment requirements, and have been evaluated in environments such as agricultural production sites, wholesale markets, and point-of-sale testing [21,44].

Several paper-based and reader-assisted platforms have been applied in field-oriented contexts for rapid detection in complex agricultural matrices with minimal reliance on external instrumentation. Zha et al. reported a dual-mode fluorescent LFA for chloroacetamide herbicides in corn that combined visual readout with intelligent quantification and a total analysis time of approximately 12 min [23]. Similarly, Bai et al. developed a nanomaterial-enhanced immunochromatographic strip for acetamiprid detection in fruits, with reported detection limits in the sub-µg/kg range using a portable reader for on-site operation [44].

Smartphone-assisted systems were applied in field-based contexts by integrating mobile imaging and software-based quantification in place of dedicated analytical readers. Chien et al. reported a plasmonic colorimetric platform that was used for on-site determination of parathion in tomato and strawberry samples using smartphone image analysis [41]. Zhao et al. employed a ratiometric upconversion nanoparticle-based paper sensor for chlorpyrifos detection in tea, with reported recovery rates exceeding 98% using smartphone-assisted image processing [22].

In contrast, several high-sensitivity platforms were classified as semi-portable or laboratory-assisted due to workflow complexity. Advanced optical platforms, such as SERS-based immunoassays and nanozyme-amplified systems, often involve controlled incubation, magnetic separation, or specialized optical components, which may limit their application in fully field-deployable settings [43]. Similarly, electrochemical platforms relying on external potentiostats were associated with the need for trained operators and stable power supply, and were therefore applied in semi-portable inspection scenarios [39].

Among the existing platforms, standalone colorimetric assays are associated with simple instrument configurations but generally exhibit higher detection limits and greater susceptibility to matrix effects. Hermanto et al. reported an AChE-based colorimetric assay for organophosphate detection in vegetables, with detection limits higher than those reported for electrochemical or immunosensor platforms [38].

Overall, the reviewed studies show that analytical sensitivity, workflow complexity, matrix tolerance, and auxiliary equipment requirements are associated with the field applicability of portable pesticide detection systems.

## 4. Discussion

### 4.1. Technological Advances in Portable Pesticide Detection

#### 4.1.1. Transition from Laboratory-Based Analysis to On-Site Screening

In the last 10 years, pesticide residue analysis has gradually shifted from laboratory-based chromatographic methods toward portable detection systems oriented for field screening. Although GC-MS and LC-MS/MS can be applied for sample analysis, their reliance on centralized laboratories, complex sample pretreatment, specialized personnel, and high operational costs limits their suitability in time-sensitive scenarios. As an alternative, most portable platforms aim to reduce analytical turnaround time and operational barriers by minimizing pretreatment steps and instrument dependence [30,40].

Early research in this area focused on miniaturizing electrochemical and enzymatic analytical methods to enable portable or semi-portable pesticide detection outside traditional laboratory environments. Studies have shown that portable electrochemical devices employing enzyme inhibition mechanisms can achieve analytically relevant sensitivity using compact potentiates instead of large analytical workstations [29,36].

In recent years, further developments in paper-based and lateral flow assay technologies have accelerated this transition, as these formats are inherently compatible with decentralized detection. Lateral flow immunoassays and test strip systems have been applied across a range of food and environmental matrices, including fruits, vegetables, soil, and water, with typical analysis times of 10–20 min [21,26]. These characteristics highlight the importance of operational simplicity and rapid response in the development of field-deployable detection technologies.

Smartphone-assisted platforms, which integrate sensing elements with mobile imaging and algorithm-assisted quantification, represent another step toward bridging laboratory analysis and field screening. These systems replace dedicated optical readers with smartphone-based data acquisition and processing, reducing hardware complexity while maintaining sensitivity levels relevant for screening purposes [20,41]. By enabling real-time interpretation of semi-quantitative results, such approaches may facilitate timely decision-making in field settings without requiring sample return or delayed confirmation.

Collectively, the literature indicates that the development of portable pesticide detection technologies is reflected not only in improvements in analytical sensitivity, but also in evolving detection scenarios and application modes.

#### 4.1.2. From Qualitative Screening to MRL-Comparable Semi-Quantitative and Quantitative Capability

Beyond improved portability, another key technological advancement is the shift in detection methods from purely qualitative screening to semi-quantitative or quantitative detection that meets regulatory maximum residue limits (MRLs). Early portable detection methods focused primarily on confirming the presence of target analytes, offering relatively limited support for decision-making in assessing regulatory thresholds. In contrast, many recent platforms explicitly report detection limits and operating ranges relative to established MRLs, reflecting growing recognition of regulatory performance requirements for field-deployable detection.

Accumulating evidence suggests that nanomaterial-assisted signal amplification has substantially improved the analytical sensitivity of portable detection platforms. Electrochemical immunosensors and affinity-based detection devices employing nanostructured electrodes or catalytic interfaces can achieve sub-microgram-per-kilogram detection limits with acceptable reproducibility, allowing for quantitative comparison with chromatographic standards [36,39].

Paper-based and lateral flow detection systems, which were traditionally regarded as qualitative technologies, have gradually evolved toward semi-quantitative formats through improved assay design and signal amplification strategies. The detection limits of lateral flow assays have approached, and in some cases fallen below, regulatory thresholds for various pesticide classes through nanoparticle-mediated amplification methods, including plasmon resonance, surface-enhanced Raman scattering (SERS), and fluorescence detection [26,44]. These improvements do not substantially increase operational complexity or testing time, suggesting their potential suitability for rapid screening contexts.

The increase in quantitative analysis capabilities of portable detection systems that is aided by an algorithm is quite pertinent in improving the analytical capacity of the systems, which is quite pronounced in the smartphone integrated platforms. Implementing optical sensors with image processing and models relying on calibration, smartphone-assisted devices may reduce user-dependent variability and improve consistency across operators and testing conditions [22,41]. These features may be particularly relevant in distributed monitoring contexts, where technical control may be more variable.

Collectively, the literature indicates that portable pesticide detection technologies are expanding beyond simple preliminary hazard identification. An increasing number of platforms are now capable of generating data directly comparable to regulatory limits, thereby supporting more informed screening decisions. While confirmatory chromatography remains irreplaceable in enforcement and legal verification, portable detection methods capable of measuring maximum residue limits (MRLs) may provide a complementary approach for integrating rapid biosensing tools into broader monitoring frameworks.

#### 4.1.3. From Single-Mode Detection to Multi-Modal and Algorithm-Assisted Readout

In recent years, the signal readout strategy of portable pesticide detection systems has gradually shifted from a single-mode approach to a detection framework that combines multiple modes with algorithm-assisted methods. Early portable biosensors primarily relied on single transduction signals, which limited their analytical stability and robustness under complex field conditions. Recent designs, by introducing complementary readout modes and algorithmic analysis, have improved the reliability and interpretability of detection results in complex food matrices.

By combining visual signals with instrument signals, dual-mode detection architectures aim to improve analytical reliability while maintaining operational simplicity. Representative lateral flow formats increasingly integrate colorimetric and fluorescence outputs, enabling both rapid visual screening and more sensitive quantitative analysis when required [21]. This redundant design may reduce the likelihood of misinterpretation through multi-signal cross-validation, thereby improving analytical robustness in field-based screening contexts.

Multimodal detection schemes have been expanded to more complex signal transduction combinations, including fluorescence–SERS systems and catalytic amplification designs. Studies have suggested that integrating orthogonal signal systems, such as combining SERS and optical readouts in lateral flow formats, may enhance detection selectivity and analytical information content without substantially increasing operational complexity [26]. Similarly, bimodal SERS-optimized immunoassays have been developed to enable rapid screening with high analytical sensitivity [32].

Another important aspect of this development is algorithm-assisted interpretation, particularly in smartphone-based systems. These platforms may reduce user-dependent variability and improve inter-test consistency by applying image analysis, calibration models, or machine learning algorithms to optical or fluorescence signals [23]. Such approaches may be particularly relevant in distributed monitoring contexts, where user conditions and environmental factors may vary.

The transition toward multimodal and algorithm-assisted detection reflects a shift in design priorities from optimizing individual signal sensitivity toward improving overall system robustness and interpretability. These developments suggest a growing emphasis on measurement reliability and cross-validation, rather than solely maximizing analytical sensitivity.

#### 4.1.4. From Proof-of-Concept Devices to Near-Product-Oriented Assay Design

Recent developments in portable pesticide biosensors have increasingly focused on the transition from proof-of-concept designs to deployable testing-kit configurations, rather than solely on improvements in analytical sensitivity or signal transduction. Early studies in this field primarily emphasized detection novelty, with limited attention to workflow standardization, reagent stability, and operator-dependent variability, which may constrain their applicability in non-controlled or field-based settings [40].

In recent years, the design of detection systems has gradually become more user-oriented, focusing on simplifying sample processing procedures, reducing operational steps, and shortening analysis time. Paper-based lateral flow systems exemplify this shift, integrating sample introduction, reaction, and signal readout into single-use kits with analysis times typically under 20 min [26,44]. Such designs may reduce user-dependent variability by simplifying operational steps, and have been explored in studies involving field-based or on-site screening scenarios.

Assay robustness and reproducibility have also received greater attention in near-product-oriented systems. Increasing attention has been given to standardized validation approaches, including recovery experiments across multiple food matrices and comparison with reference chromatographic methods, to demonstrate consistent performance under realistic testing conditions [20,32].

This is also seen in the presentation of the quantitative readouts tools that minimize subjectivity in results interpretation. Semi-quantitative or quantitative analysis becomes possible with platforms that are supported by smartphones and strip readers and remain user-friendly and allow recording and transmitting data in digital formats [22,41].

Nonetheless, the majority of the reported systems are still pre-commercial since issues of large-scale production, batch-to-batch uniformity of nanomaterials, and long-term reagent stability have not been addressed [38]. Although these developments indicate progress toward practical implementation, such systems are still primarily positioned as complementary tools rather than fully established solutions for routine monitoring.

### 4.2. Trade-Offs Between Analytical Sensitivity and Field Deployability

#### 4.2.1. Sensitivity and Operational Complexity

The literature consistently indicates that improvements in analytical sensitivity in portable pesticide detection systems are often achieved at the expense of increased operational complexity. Platforms reporting very low limits of detection frequently rely on multi-step signal amplification strategies, highly engineered nanomaterials, or tightly controlled reaction conditions, which may limit their suitability for field deployment [29,36,39].

This trade-off is particularly evident in nanozyme-enhanced immunosensor platforms. While these systems have been reported to achieve detection limits approaching or comparable to those of confirmatory chromatographic techniques, their operational requirements, including multiple reagent additions, extended incubation times, and, in some cases, partial laboratory intervention, may limit their applicability in rapid on-site screening contexts [43]. From a regulatory perspective, such very high sensitivity may exceed the requirements of first-line screening, where rapid decision-making and high throughput are often prioritized over ultra-trace quantification.

Conversely, platforms designed for field use, such as paper-based lateral flow assays (LFAs), typically sacrifice analytical sensitivity in favor of operational simplicity, rapid turnaround time, and minimal sample preparation. Although nanomaterial-assisted LFAs can achieve detection limits in sub-µg/kg ranges, their primary strength lies in their ease of use and compatibility with decentralized testing environments rather than achieving the lowest possible detection limits [21,44].

Colorimetric assays and enzyme inhibition-based systems further illustrate this trade-off. Despite having relatively higher detection limits compared to electrochemical or nanozyme-based platforms, these systems offer advantages in terms of visual readout, low instrumentation requirements, and rapid presence/absence screening. However, their performance is often influenced by matrix effects, particularly in complex food samples, which may affect signal reliability and increase the likelihood of false-positive or false-negative results [27].

Importantly, analytical sensitivity alone does not fully reflect the field applicability of a detection platform. Variability in performance across studies is not solely attributable to sensing mechanisms but is also influenced by differences in matrix complexity, validation rigor, and reproducibility. For example, enzyme-based systems may exhibit high sensitivity under controlled conditions but show reduced reliability in real matrices due to interference effects. Similarly, nanomaterial-assisted platforms may achieve low detection limits but face reproducibility challenges related to material synthesis and batch variability.

In addition, smartphone-assisted platforms, while improving accessibility and enabling semi-quantitative analysis, introduce another layer of variability related to environmental conditions such as lighting, camera quality, and user-dependent operation. These factors can affect measurement consistency and limit comparability across different testing scenarios.

Taken together, these observations suggest that the practical value of portable pesticide detection technologies is determined by a balance between sensitivity, operational complexity, reproducibility, and field adaptability, rather than the pursuit of the lowest possible detection limit. In the context of food safety monitoring, platforms that provide sufficient sensitivity within regulatory-relevant ranges, combined with operational simplicity and robustness, may be more suitable for use in routine screening contexts.

#### 4.2.2. Equipment Dependence and Field Adaptability

In addition to analytical sensitivity, equipment dependence represents a critical factor determining the applicability in field settings of portable pesticide detection technologies. The literature indicates that many high-performance platforms still rely on auxiliary devices, such as potentiostats or optical readers, which can limit their flexibility in decentralized inspection settings [29].

Although the development of portable potentiostats has reduced instrument size, electrochemical platforms still require stable power supply, standardized calibration procedures, and trained operators to ensure reliable measurements, particularly for techniques such as differential pulse voltammetry or impedance-based analysis [37]. These requirements may constrain their use in low-resource or high-throughput field environments.

A similar limitation is observed in advanced optical platforms. Fluorescence- and SERS-based systems offer high analytical sensitivity; however, their dependence on controlled excitation sources, optical alignment, and dedicated detectors may reduce their suitability for fully decentralized applications [32]. As a result, these platforms may be more appropriately positioned as semi-portable tools rather than fully field-deployable systems.

In contrast, paper-based lateral flow assays (LFAs) and reader-assisted strip platforms demonstrate clear advantages in terms of equipment independence. These systems typically require minimal or no external instrumentation and can be operated in resource-limited settings [21]. However, this operational simplicity often comes at the cost of reduced analytical precision and limited quantitative capability, highlighting a fundamental trade-off between accessibility and performance.

Smartphone-assisted systems partially mitigate the dependency on specialized equipment by leveraging widely available mobile devices for signal acquisition and analysis. While this approach enhances portability and enables semi-quantitative interpretation, it introduces new sources of variability related to camera specifications, ambient lighting conditions, and user-dependent operation [20,41]. These factors can affect measurement consistency and limit comparability across different deployment scenarios.

Importantly, equipment dependence is not solely a technical constraint but also a determinant of operational scalability. Platforms that require specialized instrumentation, calibration, or trained personnel may demonstrate strong analytical performance but encounter challenges in integration into routine monitoring workflows. In contrast, systems with minimal hardware requirements tend to be more compatible with high-throughput screening and decentralized deployment, even if their analytical sensitivity is comparatively lower.

From both regulatory and operational perspectives, field adaptability often outweighs marginal gains in analytical accuracy. Screening programs typically prioritize tools that can be rapidly deployed and easily operated by non-specialists, especially in settings with limited resources. Therefore, minimizing equipment dependence is as critical as improving analytical sensitivity for the translation of biosensor technologies into broader monitoring applications.

Overall, the evidence suggests that platforms requiring minimal hardware and operational infrastructure may have greater potential for broader implementation, whereas systems with higher equipment dependence are likely to remain more limited to controlled or semi-portable settings despite their superior analytical performance.

#### 4.2.3. Analytical Precision Versus Screening Efficiency

Another key trade-off identified in the literature is between analytical precision and screening efficiency, particularly when portable biosensor platforms are considered within real-world food safety monitoring frameworks. While confirmatory analytical methods prioritize quantitative accuracy and low uncertainty, screening systems are primarily evaluated based on throughput, turnaround time, and acceptable error rates under operational constraints [40].

From a screening perspective, achieving the lowest possible detection limit is often less critical than ensuring reliable identification of potentially non-compliant samples. In this context, minimizing false negatives is particularly important, as missed detections may lead to unsafe products entering the food supply chain. Consequently, platforms with moderate sensitivity but high throughput and rapid response times may be more practical than highly precise systems with limited processing capacity [21,27].

Improving analytical precision often requires increased assay complexity, longer incubation times, or multi-step signal amplification processes. For example, advanced optical systems such as nanozyme-enhanced immunoassays or multi-step fluorescence-based platforms typically involve extended reaction times and more complex workflows, which can reduce overall screening efficiency when large sample volumes are involved [44]. This limitation becomes particularly relevant in high-throughput monitoring scenarios.

In contrast, paper-based lateral flow assays (LFAs) and reader-assisted strip platforms are designed to prioritize rapid decision-making over high-resolution quantification. These systems enable parallel processing of multiple samples and typically deliver results within minutes, suggesting their potential suitability for rapid screening contexts [23]. Although their quantitative precision is limited, this trade-off is generally acceptable for preliminary screening purposes.

Smartphone-assisted systems partially bridge the gap between analytical precision and screening efficiency by enabling semi-quantitative analysis without substantially increasing assay complexity. However, image acquisition and data processing steps introduce additional time and potential variability, which may reduce throughput compared to visually interpreted systems and introduce user-dependent inconsistencies [42].

Importantly, analytical precision and screening efficiency should not be viewed as mutually exclusive, but rather as context-dependent performance attributes. The relative importance of each depends on the intended application, with screening programs often prioritizing speed, scalability, and reliability over absolute quantitative accuracy. In practice, the ability to rapidly identify potentially high-risk samples is often more valuable than achieving maximal analytical precision.

These considerations reinforce the role of portable biosensors as complementary tools within tiered food safety monitoring systems. Rather than replacing laboratory-based confirmatory methods, these platforms are more appropriately positioned as front-line screening tools that enable the efficient prioritization of samples for subsequent analysis.

#### 4.2.4. Platform-Specific Limitations in Real-World Applications

Beyond general trade-offs between sensitivity, complexity, and throughput, platform-specific limitations become more pronounced when biosensor technologies are applied to real food matrices. These limitations are often not fully captured under controlled laboratory conditions but significantly affect performance in practical screening environments.

Electrochemical platforms, despite their relatively high analytical sensitivity and potential for miniaturization, are particularly susceptible to electrode fouling when applied to complex food samples. Components such as lipids, proteins, and particulate matter can adsorb onto electrode surfaces, leading to signal suppression, baseline drift, and reduced reproducibility. This phenomenon has been widely reported in the electrochemical sensing of complex matrices, where surface passivation significantly affects signal stability [37,44]. This issue is especially critical in high-fat matrices, where electrode fouling may occur rapidly, requiring frequent surface regeneration or replacement.

Lateral flow immunoassays (LFAs), while highly advantageous in terms of simplicity and rapid deployment, are prone to issues related to specificity and false-positive signals. Non-specific binding, cross-reactivity with structurally similar compounds, and variability in flow dynamics can result in ambiguous test line formation, particularly near the detection threshold [21,32]. Such limitations may affect the reliability of qualitative interpretation, especially when visual readout is used without instrumental support.

Enzyme inhibition-based systems, particularly those relying on acetylcholinesterase, are inherently sensitive to matrix interference and environmental conditions. Variations in pH, temperature, and the presence of interfering substances can alter enzyme activity independently of pesticide concentration, potentially leading to false-positive or false-negative results [27,38]. This sensitivity to external conditions limits their robustness across diverse food matrices and requires careful calibration for reliable application.

Nanomaterial-assisted platforms, including nanozyme-based and SERS-enhanced systems, demonstrate excellent analytical sensitivity; however, their practical implementation is often constrained by reproducibility challenges. Variability in nanomaterial synthesis, surface functionalization, and storage conditions can introduce batch-to-batch inconsistencies, affecting signal reproducibility and limiting large-scale deployment [44].

From a practical perspective, platforms that rely on surface-sensitive detection mechanisms or complex biochemical interactions may be more vulnerable to performance degradation under field conditions. In contrast, simpler systems, although analytically less sensitive, may exhibit greater robustness in heterogeneous and resource-limited environments.

Taken together, these findings suggest that no single platform consistently outperforms others across all practical criteria. Instead, platforms that demonstrate tolerance to matrix interference, stable signal generation, and minimal dependence on controlled conditions may be more likely to achieve reliable performance in routine screening contexts.

### 4.3. Implications for Food Safety Monitoring and Regulatory Screening

The evidence synthesized in this review supports the potential use of biosensor-based rapid testing platforms as first-line tools for food safety screening, rather than as replacements for laboratory-based confirmatory analysis. In regulatory practice, such tools may facilitate rapid decision-making at critical control points by enabling the early identification of samples that may present compliance risks.

Paper-based lateral flow assays (LFAs) and reader-assisted strip platforms appear to be compatible with routine inspection workflows due to their operational simplicity, short analysis time, and minimal equipment requirements. For several organophosphorus and neonicotinoid pesticides, reported detection limits of these platforms fall within or near typical regulatory maximum residue limit (MRL) ranges (approximately 0.01–0.5 mg/kg, depending on commodity). This suggests their potential applicability for preliminary screening purposes, although they are not intended for confirmatory regulatory enforcement. Their ease of use may also support deployment by non-specialist personnel in decentralized inspection settings [21,44].

Electrochemical platforms and advanced optical assays may occupy an intermediate position within regulatory testing hierarchies. While these systems generally require portable instrumentation and a certain level of technical expertise, they offer improved analytical performance compared to simple screening tools and may support secondary screening or targeted on-site measurements to inform follow-up actions [29,35]. However, their broader implementation depends on further validation under field conditions and improved standardization.

Smartphone-assisted systems further expand decentralized screening capacity by enabling digital signal acquisition, data processing, and integration into traceability or data management systems. Nevertheless, their consistent use in routine inspection may require standardized calibration procedures and harmonized interpretation criteria, as variations in imaging conditions and device specifications can affect the analytical outcomes [20,41].

Conversely, more complex platforms including multimodal, nanozyme-based, and algorithm-driven systems remain largely at the prototype or pilot-testing stage. Although these systems often demonstrate high analytical sensitivity under controlled conditions, their operational complexity, calibration requirements, limited reagent stability, and lack of standardized validation currently may limit their applicability in routine inspection contexts.

Importantly, regulatory suitability depends not only on analytical sensitivity but also on the ability to reliably detect non-compliant samples under realistic conditions. In particular, minimizing false-negative outcomes near regulatory thresholds is critical for effective screening [9]. A platform with an ultra-low theoretical limit of detection may still be unsuitable in practice if signal variability or matrix interference increases the risk of misclassification. Therefore, rigorous validation under matrix-specific and field-relevant conditions remains essential for regulatory acceptance.

In practice, regulatory screening does not necessarily require the highest achievable analytical sensitivity at the initial testing stage. Instead, monitoring programs tend to prioritize robust, rapid, and scalable tools that can efficiently triage samples for confirmatory analysis using established chromatographic methods such as GC–MS or LC–MS/MS.

**Figure 4:**
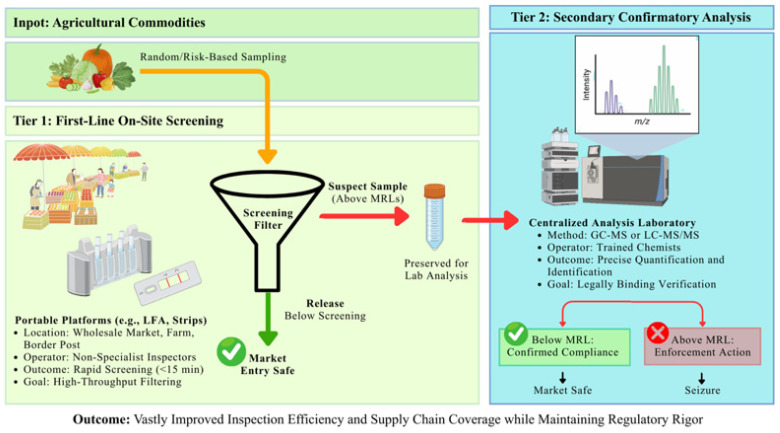
Schematic illustration of a tiered regulatory food safety monitoring framework for pesticide residue detection. Tier 1 utilizes rapid, portable biosensing platforms (e.g., LFAs, reader-assisted strips) for first-line, high-throughput on-site screening to facilitate immediate market-entry decisions. Tier 2 employs centralized laboratory instrumentation (e.g., GC-MS, LC-MS/MS) for the legally binding confirmatory analysis of suspect non-compliant samples.

Figure 4 illustrates a conceptual tiered framework for regulatory food safety monitoring, in which portable biosensors are integrated into risk-based sampling strategies. Within this framework, biosensors may be positioned as front-line screening tools used to improve inspection coverage and efficiency, particularly at high-risk points in the supply chain. Samples identified as potentially non-compliant are subsequently prioritized for laboratory-based confirmatory analysis, thereby potentially optimizing resource allocation and improving the overall monitoring effectiveness.

### 4.4. Remaining Challenges and Technology Gaps

Despite significant advancements in pesticide detection, the translation of portable biosensors into routine food safety monitoring may remain constrained by several critical challenges. These limitations extend beyond analytical sensitivity and include matrix interference, limited reproducibility, variability in operational stability, and the lack of standardized evaluation frameworks across platforms. Collectively, these factors may hinder the scalability and regulatory acceptance of biosensor-based technologies.

#### 4.4.1. Matrix Effects and Sample Complexity

Matrix effects represent one of the most significant barriers to reliable biosensor performance in real-world applications. The compositional complexity of agricultural products including pigments, lipids, sugars, organic acids, and particulate matter can interfere with signal transduction and molecular recognition processes, leading to signal suppression, enhancement, or non-specific responses [41]. As a result, analytical performance observed under controlled conditions may not be directly transferable to complex food matrices.

The literature indicates that detection performance can vary substantially across different sample types, even for the same pesticide class. For example, leafy vegetables, fruits, and processed food products may exhibit distinct matrix effects due to differences in composition and physical structure [20,41]. Although many studies attempt to address these challenges through recovery experiments or standard addition methods, such validation strategies are often limited to a small number of representative matrices [21].

Consequently, the generalizability of reported performance remains uncertain, particularly for platforms intended for broad regulatory application [39]. Current validation practices do not fully capture the diversity of food systems encountered in routine inspections, highlighting a critical gap between laboratory validation and field deployment. Addressing this issue will require more systematic evaluation of matrix tolerance across a wider range of food types and testing conditions [34].

#### 4.4.2. Standardization and Reproducibility

A major limitation in the current literature is the lack of standardized protocols for biosensor fabrication, testing, and performance reporting. While nanomaterial-assisted platforms often demonstrate high analytical sensitivity, their performance is highly dependent on synthesis conditions, surface functionalization processes, and storage history. This sensitivity can result in significant batch-to-batch variability, which complicates reproducibility across laboratories and may limit confidence in large-scale implementation [44].

In addition, key performance metrics including limit of detection (LOD), linear range, and recovery are often defined and reported inconsistently across studies. Differences in experimental design, sample preparation, and validation criteria further complicate direct comparison between platforms [32]. Without harmonized testing protocols or reference materials, it remains challenging to benchmark biosensor performance against regulatory standards in a consistent and transparent manner [36].

This lack of standardization not only affects scientific comparability but also may represent a barrier to regulatory acceptance, where reproducibility and traceability are essential requirements.

#### 4.4.3. Shelf Life and Operational Stability

Operational stability represents another critical gap between laboratory development and real-world application. Many biosensor platforms rely on biologically derived recognition elements such as enzymes, antibodies, or aptamers that are inherently sensitive to environmental conditions, including temperature, humidity, and storage duration [27]. Degradation of these components can lead to reduced sensitivity, signal drift, or complete loss of functionality.

Although nanozyme-based systems have been proposed as more stable alternatives, long-term stability data under realistic storage and field conditions remain limited [24]. Furthermore, few studies provide a systematic evaluation of shelf life, including performance over extended storage periods or under variable environmental conditions.

From a practical perspective, insufficient stability data may limit the feasibility of deploying these systems in decentralized monitoring contexts, where storage conditions may be difficult to control. Without demonstrated long-term stability and robustness, the transition from proof-of-concept devices to commercially viable assay kits may remain uncertain.

#### 4.4.4. Cross-Platform Comparability and Regulatory Alignment

Another major challenge lies in the limited comparability of analytical performance across different biosensor platforms. Many studies report limits of detection (LODs) in relation to regulatory maximum residue limits (MRLs); however, these comparisons are often based on different regulatory standards, target commodities, and experimental conditions, making an objective assessment of compliance difficult [45]. As a result, direct cross-platform comparison may be misleading if contextual differences are not adequately considered.

In addition, data interpretation and calibration procedures vary substantially across platforms. This issue is particularly evident in smartphone-assisted and algorithm-based systems, where user-defined thresholds, image acquisition conditions, and processing parameters can significantly influence reported sensitivity and signal output [42]. Such variability introduces uncertainty in performance reporting and reduces comparability between studies.

The absence of standardized calibration protocols, reference materials, and validation frameworks further complicates the translation of laboratory-based performance metrics into regulatory decision-making. Without harmonized criteria for performance evaluation, it may remain challenging to align biosensor outputs with established regulatory requirements in a consistent and transparent manner.

#### 4.4.5. Summary of Technology Gaps

Overall, the identified challenges suggest that the primary barrier to large-scale implementation of biosensor-based pesticide detection technologies is not the lack of analytical innovation, but rather insufficient alignment with the regulatory, operational, and standardization requirements.

To enable broader adoption, future development should move beyond improving analytical sensitivity alone and instead focus on: (i) systematic evaluation of matrix effects across diverse food systems; (ii) enhancement of reproducibility and long-term operational stability; and (iii) establishment of standardized performance assessment frameworks that enable consistent comparison and regulatory alignment.

Addressing these gaps may be important for supporting the transition of portable biosensors from proof-of-concept research tools to more reliable components of routine screening systems.

### 4.5. Future Directions and Research Priorities

Future studies of portable pesticide detection platforms should place greater emphasis on translational viability, rather than focusing solely on achieving higher analytical sensitivity. To provide a clearer roadmap for addressing the limitations identified in the preceding sections, Table 6 summarizes the current technology gaps alongside corresponding future research priorities.

Addressing these priorities may support the advancement of portable biosensors beyond proof-of-concept systems toward more robust and potentially scalable technologies for broader applications. One key priority is the development of multiplex detection capabilities that enable the simultaneous screening of multiple pesticide residues without substantially increasing the methodological complexity.

Although proof-of-concept studies have suggested that aptamer- or antibody-based platforms may enable multi-analyte detection, the current coverage of analytes remains limited and often requires complex operational steps. This highlights the need for simplified and scalable multiplex designs that can be more readily integrated into existing field workflows and preliminary screening contexts [23,42].

The second issue that should be addressed is the absence of unified standards for testing protocols and performance metrics. The studies reviewed revealed substantial variation in the definition of detection limits, calibration approaches, and validation methodologies, which restrict cross-platform performance comparisons and complicate interpretation in regulatory contexts. Future research may benefit from focusing on the establishment of harmonized validation frameworks, including matrix-matched calibration, consistent reporting of recovery rates, and standardized reproducibility metrics [35,40].

Stability and shelf life of sensing elements remain important considerations for long-term application. While nanozyme-based and nanomaterial-assisted systems may exhibit improved stability compared to enzyme-based platforms, the systematic evaluation of nanoparticle storage stability under practical conditions remains limited. Comprehensive stability studies may be required to support the transition from laboratory-scale systems to more robust and scalable detection formats [24,43].

Smartphone- and algorithm-based data interpretation is increasingly integrated into the design of portable detection platforms. Combining mobile imaging with machine learning-based interpretation may enhance the performance of semi-quantitative analysis. However, the validity of these approaches in regulatory screening contexts requires further evaluation and standardization. Their application in routine screening may also depend on standardized image acquisition conditions and the reduction in user-dependent variability [20,23,41].

Finally, future research should further clarify the functional role of portable biosensors in the overall food safety monitoring system, rather than using them as a substitute for laboratory confirmatory analysis. Establishing a clearer linkage between rapid on-site screening tools and confirmatory chromatographic methods may be important for improving operational efficiency and supporting regulatory reliability.

### 4.6. Limitations of This Review

This review has several limitations that should be acknowledged. First, the included studies are highly heterogeneous in terms of detection platforms, target analytes, sample matrices, and performance evaluation criteria, which limits a direct comparison across studies.

Second, a substantial proportion of the available literature consists of proof-of-concept studies conducted under controlled laboratory conditions, with relatively limited evidence from real-world or field-based applications. As a result, the practical performance and applicability of many reported platforms may not be fully established.

Third, variability in reporting practices across studies, including inconsistent definitions of key performance metrics such as limit of detection (LOD), recovery, and reproducibility, may affect the comparability and interpretation of findings.

Finally, this review is based on the available published literature, which may be subject to publication bias and uneven coverage across different types of detection technologies. These limitations should be considered when interpreting the findings and highlight the need for more standardized, application-oriented, and systematically validated research in this field.

## 5. Conclusions

This systematic review summarizes recent developments in portable biosensor technologies for the detection of pesticide residues in food and agricultural samples. Based on 26 peer-reviewed studies published between 2010 and 2025, the findings highlight the evolving roles of sensing platforms, molecular recognition strategies, and nanomaterial-assisted signal amplification in enabling rapid, field-oriented pesticide screening.

The reviewed evidence indicates a clear transition from laboratory-centered analytical approaches toward more field-adaptable detection technologies. Instead of focusing solely on achieving the lowest possible detection limits, recent developments emphasize a balanced consideration of analytical performance, operational simplicity, and suitability for on-site applications. Therefore, the evaluation of portable detection systems should extend beyond analytical sensitivity to include factors such as robustness, reproducibility, and field deployability.

Nanomaterials, including metal nanoparticles, carbon-based materials, semiconductor nanostructures, and nanozyme systems, have contributed significantly to improvements in signal stability, sensitivity, and multi-mode detection capabilities. However, several critical challenges remain, such as matrix interference, limited cross-platform comparability, insufficient long-term stability, and the lack of standardized validation frameworks. These limitations continue to restrict the broader implementation of these technologies beyond controlled experimental settings.

In the context of food safety monitoring, simpler platforms such as paper-based lateral flow assays and reader-assisted strip systems are more readily adaptable for near-term screening applications, particularly where rapid and decentralized testing is required. In contrast, more complex platforms, including multimodal, nanozyme-based, and algorithm-driven systems, remain largely at the proof-of-concept stage and require further validation before practical implementation. Therefore, these technologies should be viewed as complementary to, rather than replacements for, laboratory-based confirmatory methods. Establishing clear operational linkages between rapid on-site screening and chromatographic confirmation is crucial important for ensuring regulatory reliability and inspection efficiency.

Overall, portable biosensor technologies have established a strong technical foundation for preliminary pesticide screening in modern food systems. Their broader adoption may depend not only on continued technical innovation but also on improvements in standardization, scalability, and integration into routine monitoring workflows.

## Figures and Tables

**Table 1:** Characteristics of the 26 included studies on biosensor-based rapid pesticide detection in agricultural products.

Author (Year)	Country	Pesticide Class	Sample Matrix	Sensor Platform	Recognition Element	LOD (µg/kg)	Validation Method	Reference
Zhu et al. (2021)	China	Pyrethroid	Tea, apple, grape	Smartphone-assisted platform	MIP polymer	8.9 × 10^−1^	HPLC comparison + recovery	[20]
Zheng et al. (2024)	China	Neonicotinoid; Fungicide	River/lake water, orange juice, milk	Paper-based LFA	Antibody	2.6 × 10^−3^	ELISA comparison + recovery	[21]
Zhao et al. (2025)	China	Organophosphate; Carbamate	Apple, tomato, cucumber, leafy vegetables	Reader-assisted strip platform	AChE (enzyme inhibition)	1.5 × 10^1^	LC-MS/MS comparison + recovery	[18]
Zhao et al. (2024)	China	Organophosphate	Tea	Smartphone-assisted platform	AChE (enzyme inhibition)	1.7 × 10^0^	Recovery and RSD	[22]
Zha et al. (2024)	China; South Korea	Herbicide (Chloroacetamide)	Corn	Paper-based LFA	Antibody	8.0 × 10^−2^	Recovery and RSD	[23]
Yang et al. (2025)	China	Organophosphate	Apple, pear, river water	Paper-based LFA	AChE (enzyme inhibition)	8.5 × 10^−1^	Standard addition	[24]
Wang et al. (2025)	China	Organophosphate	Apple, tomato, spinach	Electrochemical platform	Antibody	3.0 × 10^−1^	Recovery and RSD	[25]
Sheng et al. (2021)	China	Fungicide; Insecticide; Herbicide	Soil, rice, apple, cabbage, river water	Paper-based LFA	Antibody	8.5 × 10^−2^	Recovery and RSD	[26]
Shekarchizadeh et al. (2025)	Iran	Organophosphate	Lettuce, cucumber, strawberry	Colorimetric portable platform	AChE (enzyme inhibition)	2.7 × 10^1^	Recovery and standard addition	[27]
Sahu et al. (2023)	India	Organophosphate	Tomato, potato	Reader-assisted strip platform	Host–guest binding (synthetic recognition)	4.0 × 10^0^	Recovery and RSD	[28]
Nagabooshanam et al. (2019)	India	Organophosphate	Cucumber, capsicum, brinjal	Electrochemical platform	AChE (enzyme inhibition)	6.0 × 10^−3^	Recovery and RSD	[29]
Montali et al. (2020)	Italy	Organophosphate	White cabbage juice	Smartphone-assisted platform	Enzyme cascade (multi-enzyme recognition)	1.5 × 10^1^	Recovery and RSD	[30]
Maanaki et al. (2023)	USA	Organophosphate	Juice, milk, beef, river/ ground water	Reader-assisted strip platform	AChE (enzyme inhibition)	3.0 × 10^−4^	LC-MS/MS comparison + recovery	[31]
Ma et al. (2024)	China	Neonicotinoid; Fungicide	Apple, orange	Optical/SERS portable platform	Antibody	2.7 × 10^−1^	HPLC comparison + recovery	[32]
Liu et al. (2023)	China	Fungicide (benzimidazole)	Citrus fruit, canned citrus, cabbage	Electrochemical platform	Antibody	3.7 × 10^−1^	UPLC-MS/MS comparison + recovery	[33]
Liu et al. (2012)	China	Organophosphate; Carbamate	Apple juice, tomato, cucumber, river water	Reader-assisted strip platform	AChE (enzyme inhibition)	1.0 × 10^−1^	HPLC comparison + recovery	[34]
Liu et al. (2021)	China	Organophosphate	Cabbage, tea, peach	Paper-based LFA	Aptamer (SS2–55)	2.5 × 10^0^	HPLC comparison + recovery	[35]
Li et al. (2022)	China	Organophosphate	Cabbage, leek	Electrochemical platform	BHb-based binding (affinity recognition)	1.6 × 10^0^	Recovery and RSD	[36]
Huang et al. (2025)	China	Neonicotinoid	Cucumber, cabbage, apple	Reader-assisted strip platform	Aptamer (hybrid)	8.0 × 10^−2^	Recovery and RSD	[37]
Hermanto et al. (2024)	Indonesia	Organophosphate	Onion, tomato, pepper, potato, carrot, beans, leafy vegetables	Colorimetric portable platform	AChE (enzyme inhibition)	4.0 × 10^1^	GC-MS comparison + recovery	[38]
Hatamluyi et al. (2023)	Iran	Organophosphate	Apple, lettuce, cabbage, spinach, soil, river/tap water	Electrochemical platform	Aptamer	2.4 × 10^−6^	Standard addition	[39]
Guo et al. (2015)	China	Organophosphate	Lettuce, cucumber, long bean, spinach, cabbage, bean, apple, tomato	Reader-assisted strip platform	AChE (enzyme inhibition)	1.0 × 10^−1^	Instrumental comparison	[40]
Chien et al. (2022)	China	Organophosphate	Tomato, strawberry	Smartphone-assisted platform	Plasmonic probe (signal-assisted recognition)	1.0 × 10^−1^	Real sample validation	[41]
Cheng et al. (2018)	China	Organophosphate	Spinach, lettuce, cabbage, tomato, apple, blueberry, etc.	Smartphone-assisted platform	Aptamer (multi-target)	7.3 × 10^−1^	GC-MS comparison + recovery	[42]
Chen et al. (2021)	China	Organophosphate	Cabbage, apple, pear, rice	Optical (nanozyme-enhanced) portable platform	Antibody (competitive)	1.5 × 10^−3^	LC-MS/MS comparison + recovery	[43]
Bai et al. (2022)	China	Neonicotinoid	Apple, tomato	Paper-based LFA	Antibody (monoclonal)	8.8 × 10^−3^	AuNP-ICS comparison + recovery	[44]

Note: LOD values are reported in µg/kg equivalent units to facilitate comparison across studies using different measurement formats. Sensor platforms in Table 1 are categorized based on device format and field-deployment characteristics rather than sensing mechanisms; detailed mechanistic classifications are provided in Section 3.2.

**Table 2:** Qualitative assessment of methodological robustness across the included studies.

Study	Platform	Validation Strategy	Matrix Complexity	Reproducibility	Overall Robustness	Reference
Zhu et al. (2021)	Smartphone	High (HPLC + recovery)	Real samples	Reported	High	[20]
Zheng et al. (2024)	LFA	High (ELISA + recovery)	Real samples	Reported	High	[21]
Zhao et al. (2025)	Strip	High (LC-MS/MS + recovery)	Real samples	Reported	High	[18]
Zhao et al. (2024)	Smartphone	Moderate (recovery only)	Real samples	Reported	Moderate	[22]
Zha et al. (2024)	LFA	Moderate (recovery + RSD)	Real samples	Reported	Moderate	[23]
Yang et al. (2025)	LFA	Moderate (standard addition)	Real + water	Limited	Moderate	[24]
Wang et al. (2025)	Electrochemical	Moderate (recovery + RSD)	Real samples	Reported	Moderate	[25]
Sheng et al. (2021)	LFA	Moderate (recovery + RSD)	Mixed matrices	Reported	Moderate	[26]
Shekarchizadeh et al. (2025)	Colorimetric	Moderate (recovery)	Real samples	Limited	Moderate	[27]
Sahu et al. (2023)	Strip	Moderate (recovery + RSD)	Real samples	Reported	Moderate	[28]
Nagabooshanam et al. (2019)	Electrochemical	Moderate (recovery + RSD)	Real samples	Reported	Moderate	[29]
Montali et al. (2020)	Smartphone	Moderate (recovery + RSD)	Real samples	Reported	Moderate	[30]
Maanaki et al. (2023)	Strip	High (LC-MS/MS + recovery)	Real samples	Reported	High	[31]
Ma et al. (2024)	Optical/SERS	High (HPLC + recovery)	Real samples	Reported	High	[32]
Liu et al. (2023)	Electrochemical	High (UPLC-MS/MS + recovery)	Real samples	Reported	High	[33]
Liu et al. (2012)	Strip	High (HPLC + recovery)	Real samples	Reported	High	[34]
Liu et al. (2021b)	LFA	High (HPLC + recovery)	Real samples	Reported	High	[35]
Li et al. (2022)	Electrochemical	Moderate (recovery + RSD)	Real samples	Reported	Moderate	[36]
Huang et al. (2025)	Strip	Moderate (recovery + RSD)	Real samples	Reported	Moderate	[37]
Hermanto et al. (2024)	Colorimetric	High (GC-MS + recovery)	Real samples	Reported	High	[38]
Hatamluyi et al. (2023)	Electrochemical	Moderate (standard addition)	Real samples	Limited	Moderate	[39]
Guo et al. (2015)	Strip	High (instrument comparison)	Real samples	Limited	Moderate	[40]
Chien et al. (2022)	Smartphone	Moderate (real sample validation)	Real samples	Limited	Moderate	[41]
Cheng et al. (2018)	Smartphone	High (GC-MS + recovery)	Real samples	Reported	High	[42]
Chen et al. (2021)	Optical	High (LC-MS/MS + recovery)	Real samples	Reported	High	[43]
Bai et al. (2022)	LFA	High (comparison + recovery)	Real samples	Reported	High	[44]

Note: Validation Strategy: High = chromatographic validation (LC–MS/MS, GC–MS, HPLC), Moderate = recovery/standard addition only; Matrix Complexity: buffer < spiked < real sample; Reproducibility: Reported = RSD/replicate available, Limited = not clearly reported; Overall Robustness: High = chromatographic validation + real sample, Moderate = partial validation or limited reporting.

**Table 3:** Comparison of common biorecognition elements used in portable pesticide biosensors.

Recognition Element	Recognition Mechanism	Key Advantages	Major Limitations/Field Challenges	Typical Targets
Enzymes (e.g., AChE)	Enzymatic activity inhibition	Direct functional correlation to pesticide toxicity; established commercial protocols.	High susceptibility to complex matrix effects; poor thermal stability limits shelf life.	Organophosphates, Carbamates
Antibodies (Immunosensors)	Specific antigen-antibody affinity	High specificity and selectivity; well-suited for diverse agricultural matrices.	Batch-to-batch variation; potential denaturation in harsh or fluctuating field environments.	Neonicotinoids, Fungicides, Herbicides
Aptamers (Aptasensors)	Nucleic acid structural folding/affinity binding	High chemical and thermal stability; ease of in vitro modification; lower production cost.	Performance is heavily dependent on buffer ionic strength; limited commercial availability for broad pesticide classes.	Organophosphates, Multiple targets (Multiplexing)

**Table 4:** Comparative analytical performance of portable pesticide detection platforms based on median limits of detection.

Sensor Platform	Number of Studies (n)	Median LOD (µg/kg)	Typical Validation Approach	Relative Field Deployability
Electrochemical platforms	5	0.30	LC-MS/MS, HPLC or GC comparison; recovery and RSD tests	Moderate
Paper-based platforms (LFA)	6	0.083	Recovery tests; ELISA or chromatographic comparison tests	High
Reader-assisted strip platforms	6	0.10	Chromatographic comparison; recovery and RSD	High
Smartphone-assisted systems	5	0.89	Image-based quantification; GC/HPLC comparison; recovery	High
Optical/colorimetric platforms	4	1.0 (approx.)	GC-MS or LC-MS/MS comparison; recovery tests	Very high

Note: Median LOD values were calculated using studies that could be unambiguously assigned to a single primary platform category. Each study was counted only once to avoid double counting across platform types. Multi-modal and laboratory-assisted systems were discussed qualitatively but excluded from median calculations to ensure cross-platform comparability. All reported LOD values were converted to µg/kg prior to analysis.

**Table 5:** Summary of nanomaterial-based signal amplification strategies, their functional contributions, and deployment suitability in portable pesticide detection.

Nanomaterial Category	Representative Materials	Key Properties/Mechanisms	Integrated Platforms	Primary Functional Contribution	Deployment Suitability & Limitations
Metal-Based	Gold nanoparticles (AuNPs), Metal nanostructures	LSPR, catalytic activity, high electrical conductivity	LFA, electrochemical, smartphone- assisted, SERS	Bridges the sensitivity gap between simple field assays and laboratory methods.	High to Moderate. AuNP-LFA is highly deployable, but SERS/Fluorescence requires auxiliary optical components.
Carbon & Semiconductor	Carbon dots (CDs), Upconversion nanoparticles (UCNPs)	Photostability, tunable fluorescence, ratiometric emission	Smartphone-assisted, paper-based, optical readouts	Enhances signal robustness and interpretability under varying ambient light conditions.	High. Highly compatible with smartphone imaging, though dependent on camera quality.
Nanozyme & Hybrid	Single-atom nanozymes, Bimetallic nanocomposites	Peroxidase-like catalytic activity, multi-modal capability	Enzyme inhibition assays, advanced immunoassays	Enables ultra-sensitivity and multi-modal/algorithm-assisted cross- validation.	Moderate. Complex workflows make them better suited for secondary targeted screening rather than routine field testing.

**Table 6:** Alignment of current technological gaps with proposed future research priorities for portable pesticide biosensors.

Identified Technology Gap (Challenge)	Impact on Field Deploy Ability	Proposed Research Priority (Future Direction)
Matrix Effects & Sample Complexity	Leads to false-positive/negative results; restricts generalization across diverse agricultural crops.	Develop matrix-tolerant assay architectures and mandate rigorous matrix-matched validation protocols.
Lack of Standardization & Reproducibility	Prevents direct cross-platform comparisons; severely limits regulatory acceptance and legal defensibility.	Establish unified validation frameworks and transparent reporting standards for analytical performance metrics.
Limited Shelf Life & Operational Stability	Restricts long-term storage, decentralized distribution, and overall commercial viability of field assay kits.	Conduct systematic longitudinal stability studies; integrate robust nanozymes or highly stable synthetic receptors.
Single-Analyte Focus/Low Throughput	Inefficient for comprehensive food safety screening where multiple pesticide residues frequently coexist.	Engineer scalable multiplexing capabilities without compromising operational simplicity or turnaround time.

## Data Availability

The original contributions presented in this study are included in the article/Supplementary Materials. Further inquiries can be directed to the corresponding author.

## Supplementary Materials

The following supporting information can be downloaded at: https://www.mdpi.com/article/10.3390/foods15081412/s1.
